# Brain edema after oocyte retrieval: a case report

**DOI:** 10.1186/s12905-022-02098-x

**Published:** 2022-12-12

**Authors:** Lijuan Fan, Wen Wen, Hanying Zhou

**Affiliations:** Assisted Reproduction Center, Northwest Women’s and Children’s Hospital, No.73 Houzai Gate, Xi’an, 710003 People’s Republic of China

**Keywords:** Oocyte retrieval, Hyponatremia, Brain edema

## Abstract

**Background:**

Brain edema is a rare and serious complication of assisted reproductive technology (ART). The increased intracranial pressure and injured brain parenchyma are life-threatening and may even result in death. The pathogenesis may involve increased vascular permeability mediated by vascular endothelial growth factor and other vasoactive substances, including interleukin 6, interleukin 1β, angiotensin II, insulin-like growth factor 1, transforming growth factor β, and the renin–angiotensin system.

**Case presentation:**

We presented a unique case report of a 29-year-old woman developed sudden irritability, blurred consciousness, and vomiting 8 h after oocyte retrieval. Blood examinations showed hyponatremia and cranial computed tomography showed swelling of the brain parenchyma. After therapeutic use of hypertonic saline and mannitol infusion, the patient’s consciousness recovered and her neurological state improved.

**Conclusions:**

Brain edema is a rare and serious complication of ART. Quick infusion of hypertonic salt solution and mannitol is a key treatment. A good prognosis can be achieved after prompt treatment.

## Background

With the development of assisted reproductive technology (ART), the complications associated with this technology have attracted increasing attention. Controlled ovarian hyperstimulation and transvaginal sonography (TVS)-guided ovarian follicle puncture are frequently used techniques for in vitro fertilization and embryo transfer. However, common complications of these techniques include ovarian hyperstimulation syndrome (OHSS), infection, bleeding, organ injury, and ovarian torsion. Brain edema is a rare complication of ovarian follicle puncture. The pathological phenomenon involves excessive fluid retention and increased brain tissue volume. The increased intracranial pressure and injured brain parenchyma are life-threatening and may even result in death [1]. We herein report a case of brain edema that occurred after ovarian follicle puncture in a young woman with polycystic ovary syndrome (PCOS) and analyze the possible etiology of brain edema in such cases.

## Case presentation

A 29-year-old woman had been infertile for 3 years and was diagnosed with PCOS at Northwest Women’s and Children’s Hospital. Her menses had been abnormal since the onset of menarche at the age of 13 years, occurring at 28–90-day intervals. She had no history of hirsutism, hyperandrogenism, cardiovascular disease, glucose metabolic diseases, or craniocerebral diseases. Her weight was 58 kg, height was 164 cm, and blood pressure was 140/80 mmHg. Her total antral follicle number was 24. Her serum anti-müllerian hormone concentration was 5.02 ng/mL, basal follicle-stimulating hormone was 4.99 IU/L, basal luteinizing hormone was 15.08 IU/L, and testosterone was 64.0 ng/dL.

After pituitary down-regulation with 3.75 mg of triptorelin acetate (IPSEN, Paris, France), the patient was started on 150 IU of human menopausal gonadotropin (menotropin) daily (Ferring Pharmaceuticals, Saint-Prex, Switzerland). This dose was continued for 8 days, after which it was increased to 175 IU daily for an additional 4 days. Recombinant human chorionic gonadotropin (250 µg; Serono Laboratories, Randolph, MA, USA) was administered when two follicles of > 18 mm were noted. The patient’s serum estradiol concentration reached 14,568 pg/mL on the day of human chorionic gonadotropin administration. She had no obvious discomfort such as abdominal distension, abdominal pain, nausea, or vomiting before oocyte retrieval. Additionally, the patient’s routine blood examination findings, liver function indices, and plasma D-dimer concentration were normal at that time.

Oocyte retrieval was performed 36 h later by TVS-guided aspiration with intravenous injection of propofol. Before oocyte retrieval, the size of the right and left ovary had increased to 62 × 56 mm and 58 × 55 mm, respectively. Thirty oocytes were obtained, and the operation time was 10 min. No fresh bleeding occurred at the vaginal puncture point after the operation. The patient’s heart rate, respiration, blood oxygen saturation, and blood pressure were normal after awakening from anesthesia. Her Steward score reached 6 before she left the operating room.

**Fig. 1:**
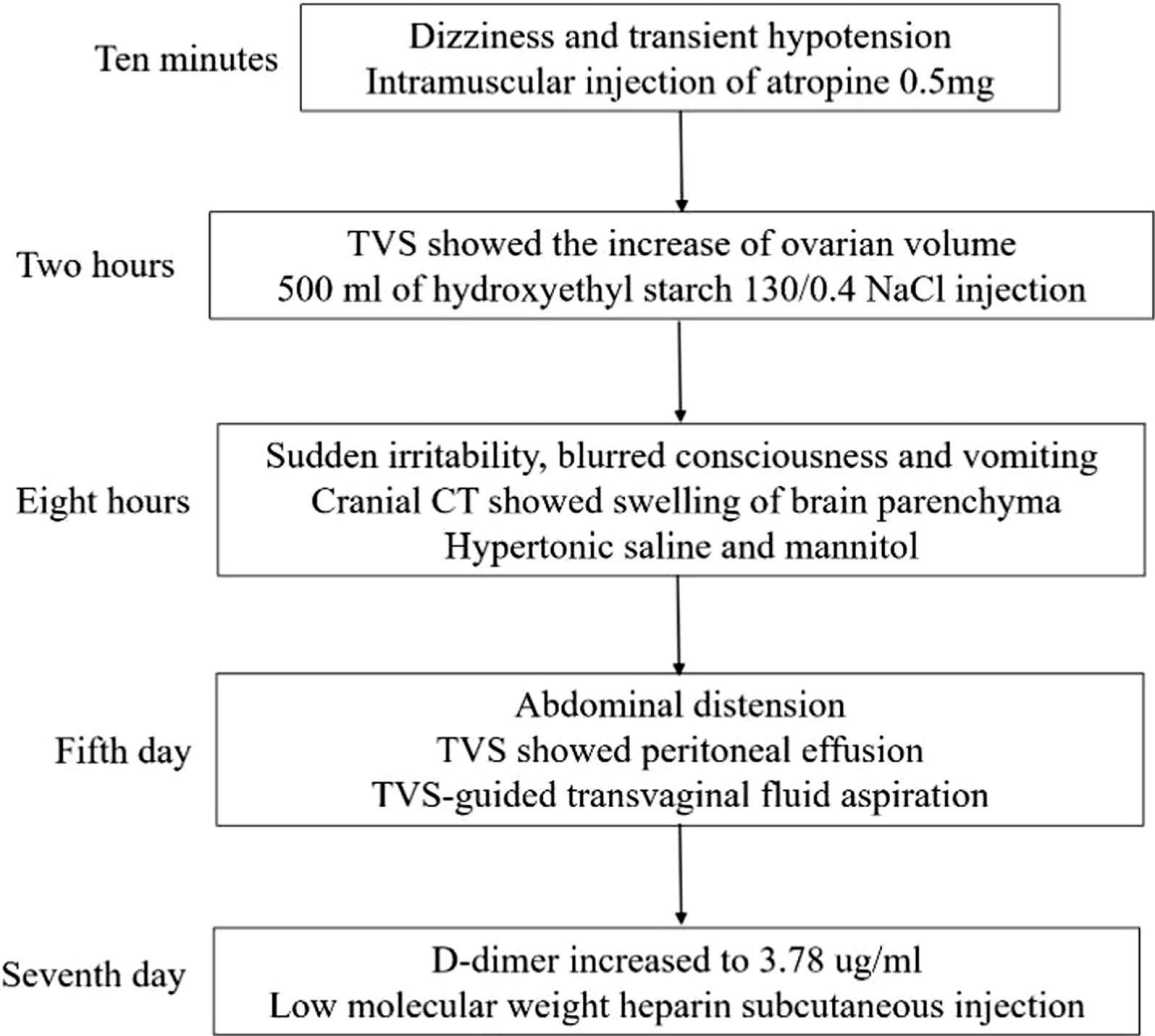
Flow chart of complications. *TVS* transvaginal sonography, *CT* computed tomography

The patient developed dizziness and transient hypotension 10 min after oocyte retrieval, and her blood pressure decreased to 80/60 mmHg. After intramuscular injection of 0.5 mg of atropine (Runhong Pharmaceutical, Henan, China), the patient’s symptoms improved and her blood pressure returned to normal (Fig. 1).

Two hours after oocyte retrieval, TVS showed that the size of the left and right ovary was 108 × 98 mm and 99 × 98 mm, respectively, and that the size of the pelvic cavity was 30 × 26 mm. Routine blood examination (Table 1) showed that her leukocyte count had increased and her hematocrit had decreased. As a routine preventive measure against OHSS, the patient received 500 mL of hydroxyethyl starch 130/0.4 in NaCl injection (Beijing Fresenius Kabi Pharmaceutical, Beijing, China).

**Table 1:** Dynamic changes in blood biochemical indices and D-dimer concentration after oocyte retrieval

Time	White blood cell	Hematocrit	Total protein	Albumin	K+	Na+	D-dimer
Reference	4–10 × 10^9^/L	36–50 L/L	60–82 g/L	34–55 g/L	3.5–5.3 mmol/L	136–145 mmol/L	0–2.0 ug/ml
Two hours	19.39	33.5	–	–	–	–	0.32
Eight hours	15.76	27.8	56.4	32.8	3.3	119.4	–
Day 1	16.75	33.5	–	–	–	–	0.37
Day 2	–	–	57.73	33.7	4	132.9	–
Day 4	18.62	–	–	–	3.9	125.9	–
Day 7	16.71	38.1	42.19	23.9	4.3	129.1	3.78
Day 11	9.66	33.2	51.92	28.9	4.1	131	4.65

**Fig. 2:**
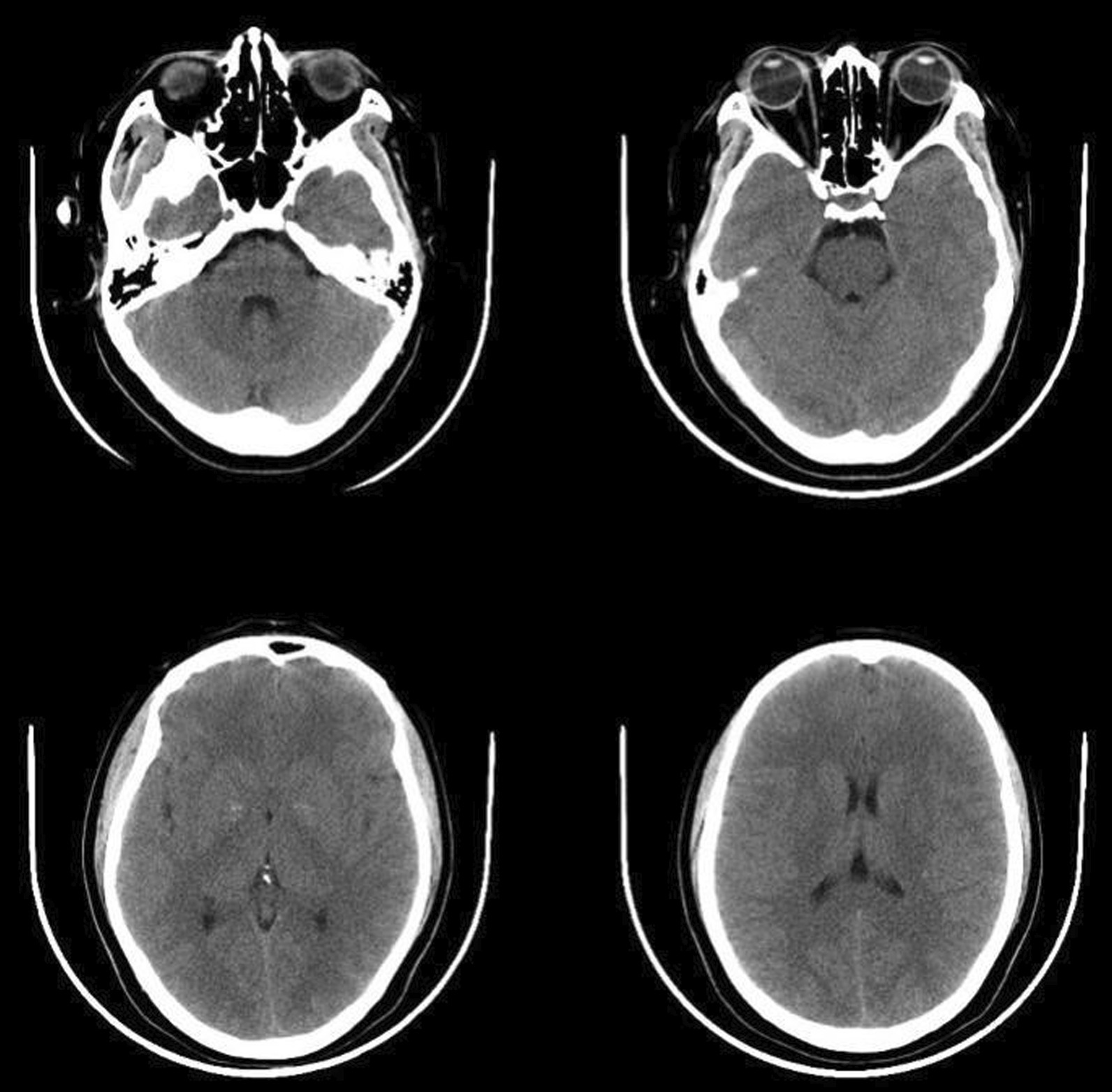
Swelling of brain parenchyma (cranial computed tomography)

The patient developed sudden irritability, blurred consciousness, and vomiting 8 h after oocyte retrieval. Her blood pressure decreased to 110/70 mmHg, pulse rate was 75 beats/min, and blood oxygen saturation was 98%. Her serum sodium concentration was 119.4 mmol/L and serum potassium concentration was 3.3 mmol/L. Cranial computed tomography showed swelling of the brain parenchyma (Fig. 2). Based on the results of serum electrolyte measurement and cranial computed tomography, the patient was treated with 250 mL of an intravenous drip of 3% NaCl combined with oral sodium supplementation and 125 mL of a rapid intravenous drip of 20% mannitol injection. She recovered 12 h later.

On the fifth day of hospitalization, the patient developed abdominal distension. TVS showed peritoneal effusion with a depth of 42 mm in the right lower abdomen, 79 mm in the lateral abdomen, 15 mm in the hepatorenal space, 22 mm in the perihepatic region, 44 mm in the left lower abdomen, 45 mm in the left abdomen, 9 mm in the splenorenal recess, 15 mm in the perisplenic region, and 68 mm in the pelvis. After TVS-guided transvaginal fluid aspiration, 4000 mL of liquid was removed.

On the seventh day of hospitalization, the patient’s serum D-dimer concentration increased to 3.78 µg/mL. She was treated with 5000 IU of low-molecular-weight heparin subcutaneous injection each day. On the 14th day of hospitalization, the patient was cured and discharged from the hospital. Informed consent for publication was obtained from the patient.

## Discussion and conclusions

Brain edema is a rare complication following ART with life-threatening consequences. It is a fatal pathological state in which the brain volume increases as a result of abnormal accumulation of fluid within the cerebral parenchyma. The expanded cerebral tissue and elevated intracranial pressure may induce adverse conditions including reduction of cerebral blood flow, hypoxia, high pressure of the cerebral tissue, and hernia formation. Brain edema is mainly classified as vasogenic edema and cytotoxic edema. The patient in the present report had no history of craniocerebral or cardiovascular disease. Cytotoxic edema is characterized by intracellular accumulation of fluid and sodium, resulting in cellular swelling. Our patient’s serum sodium concentration decreased to 119.4 mmol/L, at which time she developed sudden irritability, blurred consciousness, and vomiting. Hyponatremia may have been the primary cause of our patient’s brain edema after oocyte retrieval.

Hyponatremia develops secondary to a primary sodium deficit, primary potassium deficit, primary water excess, or a combination of these conditions. Arteriolar vasodilation and rising capillary permeability are classic physiologic changes after controlled ovarian hyperstimulation and oocyte retrieval and are also the pathophysiological mechanisms underlying OHSS [2]. Fluid shifts from the intravascular to extravascular spaces, resulting in pleural effusion, pericardial effusion, and peritoneal effusion. Vascular endothelial growth factor (VEGF) appears to mediate vascular permeability [3]. Other systemic and local vasoactive substances, including interleukin 6, interleukin 1β, angiotensin II, insulin-like growth factor 1, transforming growth factor β, and the renin–angiotensin system are also directly and indirectly involved in the pathogenesis of extravascular fluid shifts [4].

Reduced sodium intake may have been a secondary cause of hyponatremia and brain edema in the present case. Preoperative fasting for 8 h and anorexia after awakening from anesthesia may have resulted in reduced sodium intake.

Rapidly developing hyponatremia causes brain edema, and the risk of cerebral herniation is the most concerning issue. Death or profound neurologic injury has been reported when acute hyponatremia was not corrected immediately. Acute hyponatremia can be corrected more rapidly than chronic hyponatremia because in acute hyponatremia, the process of extrusion of organic osmolytes facilitated by the brain volume regulatory response has not yet taken full effect. Hypertonic saline is the mainstay of treatment for symptomatic hyponatremia because a rising serum sodium concentration reduces brain edema [5]. In the present case, the patient was treated with an infusion of 250 mL of 3% NaCl at the onset of symptomatic hyponatremia. Moreover, oral salt loading may increase the serum sodium concentration to some extent. Overly aggressive correction of the serum sodium concentration can result in neurological injury caused by osmotic demyelination. Administration of mannitol can reduce intracranial pressure and prevent cerebral herniation. The patient’s consciousness recovered and her neurological state improved after treatment with hypertonic saline and mannitol.

D-dimers are fibrin degradation products that have high sensitivity for thromboembolic disease. The factors associated with thromboembolic disease after in vitro fertilization and embryo transfer include serum estradiol, VEGF, and “third-spacing” of body fluid. Although thromboembolic complications after OHSS are rare, they may lead to critical neurological or cerebrovascular damage. Therefore, our patient was treated with low-molecular-weight heparin to prevent thromboembolic disease.

Brain edema is a rare and serious complication of ART. The pathogenesis may involve increased vascular permeability mediated by VEGF and other vasoactive substances, including interleukin 6, interleukin 1β, angiotensin II, insulin-like growth factor 1, transforming growth factor β, and the renin–angiotensin system. Excessive panic is unnecessary if brain edema occurs after oocyte retrieval. The key treatment is quick infusion of hypertonic salt solution and mannitol, and a good prognosis can be obtained after prompt treatment.

## Data Availability

All data for the case reports are available in this manuscript.

## Abbreviations

OHSS: Ovarian hyperstimulation syndrome
TVS: Transvaginal sonography
VEGF: Vascular endothelial growth factor
